# Ex-Gaussian, Frequency and Reward Analyses Reveal Specificity of Reaction Time Fluctuations to ADHD and Not Autism Traits

**DOI:** 10.1007/s10802-018-0457-z

**Published:** 2018-07-19

**Authors:** Nicoletta Adamo, John Hodsoll, Philip Asherson, Jan K. Buitelaar, Jonna Kuntsi

**Affiliations:** 1 Social, Genetic and Developmental Psychiatry Centre, Institute of Psychiatry, Psychology and Neuroscience, King’s College London, De Crespigny Park (PO80), London, SE5 8AF UK; 2 Department of Biostatistics & Health Informatics, Institute of Psychiatry, Psychology and Neuroscience, King’s College London, De Crespigny Park (PO20), London, SE5 8AF UK; 3 Department of Cognitive Neuroscience, Donders Institute for Brain, Cognition and Behaviour, Radboud University Medical Centre, Trigon building, Route 200, Kapittelweg 29, 6525 EN Nijmegen, The Netherlands

**Keywords:** Autism, ADHD, Reaction-time variability, Reward sensitivity

## Abstract

Both attention-deficit/hyperactivity disorder (ADHD) and autism spectrum disorder (ASD) have been linked to increased reaction time variability (RTV), a marker of attentional fluctuation. Here we test whether specificity to either trait emerges when we examine (1) detailed ex-Gaussian and frequency RTV subcomponents, (2) effects while controlling for the other trait and (3) improvement in the RTV measures following rewards or a faster event rate. 1110 children aged 7–10 years from a population-based sample completed a Go/No-Go task under three conditions (slow, fast and incentives). We measured RTV with standard deviation of RT (SDRT), ex-Gaussian distribution measures (Sigma and Tau), RT fluctuations in cycles of ~14–90 s in all conditions (Slow-4 and Slow-5), and RT fluctuations in cycles of 2–14 s in the fast condition (Slow-2 and Slow-3). Parent-rated ADHD and ASD traits were obtained. All refined RTV components were linked to ADHD traits only and not to ASD traits, while Sigma did not relate to either trait. Although both ADHD and ASD social-communication traits were associated with SDRT, the association with social-communication impairments disappeared when controlling for ADHD traits. A reward-induced improvement in RTV measures, indicating malleability, emerged in relation to ADHD traits but not ASD traits. Under closer inspection, specificity emerges of high RTV to ADHD traits. For the clinician, our findings indicate that attentional fluctuation in children with high ASD traits may be due to co-occurring ADHD traits and emphasise how the effectiveness of rewards does not generalise from ADHD to ASD traits.

**Electronic supplementary material:**

The online version of this article (10.1007/s10802-018-0457-z) contains supplementary material, which is available to authorized users.

## Introduction

Attention-deficit/hyperactivity disorder (ADHD) and autism spectrum disorders (ASD) show high rates of co-morbidity and significant overlap in genetic influences (Rommelse et al. 2010) and in several neurocognitive impairments, including those in executive functioning, sustained attention, and response to rewards (Rommelse et al. 2011). Of individual measures that show an association with both ADHD and ASD, reaction time variability (RTV) – linked to the neural mechanisms underlying attention allocation (Cheung et al. 2017) and arousal regulation (James et al. 2016), − is a particularly promising candidate for investigation that might ultimately inform the neurobiology of the two disorders and their overlap. Accordingly, here we test whether RTV measures may help find common and unique impairments in ADHD and ASD under varying task conditions.

In ADHD research, high RTV has emerged as one of the neurocognitive impairments showing the strongest phenotypic and genetic association with the diagnosis and with continuous ADHD symptom scores (Kofler et al. 2013; Kuntsi et al. 2010; Crosbie et al. 2013). Evidence is now also accumulating of a phenotypic association of high RTV with the ASD diagnosis and traits (Karalunas et al. 2014; Pinto et al. 2016), which is partly explained by shared genetic influences (Pinto et al. 2016). Given the high co-occurrence of ADHD and ASD, a further question is whether the association of high RTV with ASD may be explained by co-occurring ADHD symptoms. By pooling the results of 17 studies, Karalunas et al. (2014) obtained tentative evidence that, although high RTV is indeed associated with ASD, this is only in the presence of comorbid ADHD. This is further consistent with data from a general population study suggesting that RTV may predict ADHD traits beyond ASD traits (Truedsson et al. 2015).

Commonly, investigators have measured RTV with the standard deviation of RTs (SDRT). However, SDRT represents an overall phenomenon; to better understand the association of ADHD and ASD with RTV, it may be informative to decompose SDRT into its components that capture the extremely slow RTs within the individual’s performance (Leth-Steensen et al. 2000) or the periodic dynamics of their RTs (Castellanos et al. 2005). One such approach is the ex-Gaussian analysis, which separates RT distributions into their normal (Gaussian) and exponential (ex-Gaussian) parts. Analyses on RT data from participants with ADHD indicate that a set of infrequent, ultra-long RTs (the ex-Gaussian Tau) specifically contribute to increased RTV (Hervey et al. 2006). So far, two studies have directly compared children with ADHD and those with ASD in relation to ex-Gaussian parameters: in one study elevated Tau characterised ASD regardless of the co-occurrence of ADHD (Geurts et al. 2008), while the other study found elevated Tau in children with ADHD only and those with comorbid ADHD and ASD (Tye et al. 2016). The shorter task duration (3-min) in the first study (Geurts et al. 2008) might not have captured slower patterns of responses typically observed in ADHD in longer tasks such as that in the second study (~9-min) (Tye et al. 2016). Thus, experimental conditions might explain inconsistencies between studies.

A second approach uses frequency decompositions of RT data to identify the periodic patterns of RTV (e.g., cycles of ≥5 s) (Castellanos et al. 2005; Feige et al. 2013; Johnson et al. 2007). An early small study that examined slow RT fluctuations (15–20-s cycles) reported that increased RTV in these slow frequencies occurs in ASD groups and differentiates children with comorbid ASD-ADHD from those with ADHD only (Geurts et al. 2008). More recent data suggest that elevated RT fluctuations in a wide range of frequencies may be specifically associated with ADHD and not with ASD, and that RT fluctuations occurring in relatively rapid cycles (2–5-s) are elevated in children with ASD who show high ADHD symptoms (Adamo et al. 2013). Altogether, findings from ex-Gaussian and RT-fluctuation analyses suggest that RTV subcomponents may help find common and unique RTV profiles in ADHD and ASD, and ultimately guide the understanding of their underlying mechanisms.

Beyond the study of the RTV subcomponents, another approach that may inform on the underlying mechanisms of both disorders and their overlap is the investigation of the malleability of RTV impairments. Studies on population (Kuntsi et al. 2009, 2005) and clinical (Andreou et al. 2007; Epstein et al. 2011; Hervey et al. 2006) samples converge in indicating that task manipulations with rewards and faster stimulus presentation rates, alone or in combination, elicit greater RTV improvements in children with ADHD than in control children, and in relation to continuously measured ADHD symptoms. By directly comparing children with ADHD, children with ASD and those with co-morbid ADHD-ASD to control children, Tye et al. (Tye et al. 2016) found that greater improvements of overall and ex-Gaussian RTV measures in a fast-incentive condition were observed in the ADHD-only and co-morbid ADHD-ASD groups, and not in the ASD-only or control groups, suggesting that the RTV malleability could be specific to ADHD.

Overall, the available results on detailed RTV measures and their sensitivity to task manipulations have emerged from relatively small samples and mostly from clinical populations; no study to date has examined these detailed phenotypes in relation to both ADHD and ASD traits in non-clinical samples. Applying these analyses in an unselected general population sample avoids possible selection biases associated with clinic-referred or selected community samples, and provides a tool to capture detailed RTV impairments in relation to the full spectrum of ADHD and ASD symptoms. Further, examining ADHD and ASD traits is in line with recent proposals for transdiagnostic, neurobiologically grounded features that underlie the aetiology of psychopathology (Morris and Cuthbert 2012). Identifying the links of such features beyond the diagnoses of ADHD or ASD may therefore help further understand the underlying mechanisms of the observed clinical overlap between the disorders.

Here, we aim to extend initial reports of common and disorder-specific refined RTV components in ADHD and ASD in a large-scale study on a population sample of children. We perform frequency and ex-Gaussian decompositions of RT data, which previously indicated positive associations of overall RTV with the inattention and hyperactivity-impulsivity subdomains of ADHD, as well as with ASD social-communication difficulties (Kuntsi et al. 2009; Pinto et al. 2016). We first aim to investigate which frequency and ex-Gaussian RTV subcomponents are associated with ADHD symptoms (inattention and hyperactivity-impulsivity) and which with ASD symptoms (social-communication difficulties and repetitive-restricted behaviours and interests), using a ‘baseline’ slow task condition and a faster condition that allows measuring RTV indices in a range of slow and fast patterns. We then test whether the association of one trait with each RTV measure remains when controlling for both subdomains of the other trait, and whether ASD and ADHD symptoms have additive effects on RTV increases. Finally, we aim to investigate whether the RTV subcomponents’ malleability (improvement with faster event rate or incentives) differentiates between ADHD and ASD traits.

## Methods

### Sample and Procedure

Participants are members of the Study of Activity and Impulsivity Levels in children (SAIL) (Kuntsi et al. 2006), a general population sample of twins aged 7–10 years. Sampling methods and data collection procedures are described in detail in Supplement 1, available online. The parents of all participating children provided informed consent, with ethical approval obtained from the Research Ethics Committee of the Institute of Psychiatry, King’s College London, UK. The final sample consisted of 1312 individuals: 257 monozygotic (MZ) twin pairs, 181 same-sex dizygotic (DZ) and 206 opposite-sex DZ twin pairs, as well as 24 singletons coming from pairs with one of the twins excluded. The mean age of the sample was 8.83 years (SD = 0.67), and 51% of the sample were girls.

### Measures

#### Rating Scales for ADHD and ASD Traits

##### ADHD Traits

Parents completed the Long Versions of Conners’ Parent Rating Scales (Conners 1997) when children participated in SAIL. Here, we used the sum of the parent ratings on the DSM-based 9-item inattention (ADHD-I) and 9-item hyperactivity-impulsivity (ADHD-HI) subscales. Parent ratings were missing for 2 children.

##### ASD Traits

Parents also completed the Childhood Autism Spectrum Test (CAST) (Scott et al. 2002; Williams et al. 2005) when children were aged eight, approximately one year after the cognitive assessments and the completion of the Conners’ Parent Rating Scales. The CAST is a 30-item, dichotomous (yes or no) response scale. A sum of ≥15 is the cut-off for identifying children at risk for ASD (Scott et al. 2002). The items were designed to address all three domains of impairments of DSM-IV-TR-defined ASD: social impairments, communication impairments, restrictive and repetitive behaviours and interests. Using a validation sample of children clinically assessed using the Autism Diagnostic Interview–Revised and Autism Diagnostic Observation Schedule, the CAST has been shown to have good sensitivity (100%) and specificity (97%) as a screening instrument (Williams et al. 2005). The CAST demonstrated good test–retest reliability (*r* = 0.83) (Williams et al. 2006) and, in a general population sample of twins which also included our participants, the CAST displayed adequate overall internal consistency (α = 0.73) (Hoekstra et al. 2010). We divided the 37-items of the CAST into two subscales: a social-communication impairments (SCI) subscale, which consisted of 24 items, and a 7-item subscale on restricted-repetitive behaviours and interests (RRBI), according to the DSM-5 criteria (American Psychiatric Association 2013). In our sample, based on Kuder-Richardson 20 test, the internal consistency was considered acceptable for the SCI subscale (α = 0.75) and satisfactory for the RRBI subscale (α = 0.57). CAST ratings were not obtained for all TEDS cohorts and were therefore missing for 162 SAIL participants. Only participants with complete ADHD and ASD data were included in our analyses (*n* = 1148).

#### The Go/No-Go Task (Borger et al. 1999; Kuntsi et al. 2005; Van der Meere et al. 1995)

On each trial, one of two possible stimuli appeared for 300-ms in the middle of the computer screen. Children were instructed to respond only to the ‘Go’ stimuli (the letter X) and to react as quickly as possible, but to maintain a high level of accuracy. The proportion of ‘Go’ to ‘No-Go’ trials was 4:1. The participants performed the task under three conditions (slow, fast and incentive), matched for length of time on task. Seventy-two trials were presented with a fixed inter-stimulus interval of 8-s for the slow and incentive conditions; the fast condition, with an inter-stimulus interval of 1-s, consisted of 462 trials. The order of presentation of the slow and fast task conditions varied randomly across participants, whilst the incentive condition was always administered last. In the incentive condition, each correct response to the letter X and each correct nonresponse to the letter O earned the child 1 point. The child lost 1 point for each omission error (failure to respond to X) and for each failure to respond within 2-s. Each commission error (incorrect response to O) led to the loss of 5 points. The points were shown in a box, immediately right of the screen center, and were updated continuously throughout. The child started with 40 points to avoid the possibility of a negative tally. The child was asked to try to win as many points as possible and was told that the points will be exchanged for a real prize when the game ends. Due to technical issues, data were not available for 5, 6 and 7 children for the slow, fast and incentive conditions, respectively.

RT data were included from children with accurate and plausible (>150-ms) responses on ≥70% of Go trials in the slow condition and on ≥70% of Go trials in either the fast or the incentive condition, to only include participants with sufficient engagement in the task. The final sample consisted of 1110 children who had data from the slow condition. Among these, 1077 also had data from the fast condition, and 1105 also from the incentive condition. Table 1 displays the mean raw score, SD, and range for each of the ADHD and ASD traits for the included participants.

**Table 1:** Mean score on ADHD and ASD traits for the included participants (*n* = 1110)

	Mean	SD	Range
Inattention	6.00	5.45	0–27
Hyperactivity-impulsivity	6.00	5.08	0–27
Social-communication Impairments	3.24	2.67	0–21
Restricted-repetitive Behaviours and interests	1.34	1.25	0–7

#### RTV Measures

##### Overall RTV

For each dataset included in the analysis, we calculated the overall variability as the standard deviation of RT (SDRT).

##### Ex-Gaussian Parameters

We applied ex-Gaussian deconvolution to RT data employing a maximum-likelihood algorithm (Heathcote et al. 2004), implemented in the QMPE software (http://newcl.org/software/qmpe.htm). This algorithm measures the mean of the normal component of the RT distribution (mu) and divides the RTV into its normal (Sigma) and exponential (Tau) components. Here, we investigated the variability components Sigma and Tau.

##### RT Fluctuations

We further characterised RTV using frequency decomposition. To obtain continuous RT time-series for each participant, we interpolated all ‘No-Go’ datapoints, missing and anticipatory responses by replacement with the average of adjacent RT datapoints. Given the pseudo-randomized sequence of trials, we controlled for potential effects of sequence on RTV applying a linear regression that yielded residual RT time-series. We then applied the Fast-Fourier Transform to each participant’s residual time-series as previously described (Adamo et al. 2012; Johnson et al. 2007), to obtain the power spectrum, which reflects the magnitude of the signal –i.e., the variation in RTs over time– and is calculated as the squared amplitude of that signal within a frequency band. We quantified the power of frequencies, averaged over time, in the 0–0.063 Hz interval for the slow and incentive conditions, and the 0–0.5 Hz interval for the fast condition. The power spectrum was divided into bands a priori identified based on physiological models of brain oscillations (Penttonen and Buzsáki 2006): the Slow-5 (0.010–0.027 Hz) and Slow-4 (0.027–0.073 Hz) frequency bands in all conditions, and the Slow-3 (0.073–0.2 Hz) and Slow-2 (0.2–0.5 Hz) bands captured by the fast condition. RT fluctuations at frequencies included in the Slow-5 range occur in cycles of ~55-s, those included in Slow-4 occur in cycles of ~20-s, while the Slow-3 and Slow-2 ranges correspond to cycles about once every 10- and 4-s, respectively.

### Statistical Analyses

To account for positive skewness, we applied square root-transformation to Sigma and log-transformed all remaining measures prior to analysis, following the best approach suggested for each measure by the *gladder* function in Stata. Centering of RTV measures, ADHD and ASD traits was applied before analysis. For measures examined in all three conditions (SDRT, Sigma, Tau, Slow 5 and Slow 4), we evaluated the relationships between each RTV measure and each trait, as well as the effect of task condition, using a series of linear mixed-effects model analyses. Specifically, we modelled each RTV measure as a function of the participant’s rating on the ADHD or ASD trait, the task condition as a categorical factor and their interaction. To account for the non-independence of observations originating from within the same family (i.e., twin pairs) and expected within-subject correlations among datapoints collected from the same individual, the mixed models included random effects for participants and family. For analyses on the additional Slow-3 and Slow-2 frequency bands captured only by the fast condition, we used separate linear regression models (while controlling for family relatedness). Because the analyses were carried out using standardized scores, the β coefficients resulting from the regression models represent a standardized effect size measure such that a 1–standard deviation change in the ADHD/ASD trait leads to β change in standard deviation in RTV. The effect size is comparable to that of correlation coefficients.

To account for developmental effects and potential sex differences (Dykiert et al. 2012), we included age and sex as covariates. To control for multiple testing in our analyses, a false discovery rate (FDR) correction was used (Benjamini and Hochberg 1995), based on an alpha <0.05. Results are reported along with the FDR-adjusted *p* values. Analyses were repeated to model the association between each measure and trait by controlling for the effect of both subdomains of the other trait, thus testing their specific associations with the measure. Second, when both ADHD and ASD symptoms were associated with a RTV measure or its change across conditions, we planned a hierarchical regression model for each RTV measure showing an association with both traits; by adding one of the two traits first, and the other trait second, we tested whether the addition of the second trait significantly improved the prediction of RTV beyond the first trait.

## Results

### Associations of RTV Measures with ADHD and ASD Traits

Both ADHD traits significantly predicted all RTV detailed measures examined in the slow condition, except for Sigma, and those examined in the fast condition (Table 2). No significant associations emerged for either ASD trait with Sigma, Tau, Slow-5 or Slow-4 RT fluctuations in the slow condition, or the Slow-3 and Slow-2 RT fluctuations in the fast condition (Table 2). Both ADHD traits and SCI, but not RRBI, significantly predicted SDRT in the slow condition (Table 2), as reported previously for RT data combined across different tasks in this sample (Pinto et al. 2016).

**Table 2:** Predictive effects of each ADHD and ASD trait on the RTV measures in the slow condition and those captured only by the fast condition

	ADHD-I		ADHD-HI		SCI		RRBI	
	β [95% CI]		β [95% CI]		β [95% CI]		β [95% CI]	
	Crude	Controlling for ASD traits	Crude	Controlling for ASD traits	Crude	Controlling for ADHD traits	Crude	Controlling for ADHD traits
Slow condition								
SDRT	0.14 [0.9, 0.20]***	0.14 [0.08, 0.19]***	0.11 [0.06, 0.17]***	0.10 [0.05, 0.16]***	0.07 [0.02, 0.13]*	0.05 [−0.01, 0.10]	0.03 [−0.03, 0.08]	0.02 [−0.04, 0.07]
Sigma	0.05 [−0.01, 0.11]	0.06 [0.00, 0.12]	0.01 [−0.04, 0.07]	0.02 [−0.04, 0.08]	−0.02 [−0.10, 0.02]	−0.06 [−0.12, 0.00]	−0.03 [−0.09, 0.03]	−0.04 [−0.09, 0.02]
Tau	0.15 [0.09, 0.21]***	0.14 [0.09, 0.20]***	0.08 [0.02, 0.13]***	0.07 [0.01, 0.12]	0.05 [−0.00, 0.11]	0.03 [−0.03, 0.09]	0.03 [−0.03, 0.09]	0.02 [−0.04, 0.08]
Slow-5	0.13 [0.08, 0.18]***	0.12 [0.07, 0.17]***	0.11 [0.06, 0.16]***	0.11 [0.05, 0.16]***	0.03 [0.00, 0.10]	0.03 [−0.03, 0.08]	0.04 [−0.01, 0.09]	0.02 [−0.03, 0.08]
Slow-4	0.13 [0.08, 0.19]***	0.13 [0.07, 0.18]***	0.11 [0.05, 0.16]***	0.10 [0.04, 0.15]***	0.06 [0.00, 0.11]	0.03 [−0.02, 0.09]	0.03 [−0.02, 0.08]	0.02 [−0.04, 0.07]
Fast condition								
Slow-3	0.15 [0.09, 0.21]***	0.14 [0.08, 0.21]***	0.12 [0.06, 0.18]***	0.11 [0.05, 0.18]***	0.06 [0.00, 0.12]	0.01 [−0.05, 0.08]	0.04 [−0.02, 0.11]	0.02 [−0.05, 0.08]
Slow-2	0.14 [0.09, 0.19]***	0.14 [0.08, 0.20]***	0.12 [0.06, 0.18]***	0.12 [0.05, 0.18]***	0.06 [0.00, 0.12]	0.01 [−0.05, 0.07]	0.05 [−0.01, 0.11]	0.02 [−0.04, 0.08]

*ADHD-I*, inattention; *ADHD-HI*, hyperactivity-impulsivity; *RRBI*, repetitive-restricted behaviours and interests; *SCI*, social-communication impairments; *SDRT*, standard deviation of RT

*p < 0.05; **p < 0.01; ***p < 0.001

With the effect of the ASD traits removed in post-hoc analyses, the association of ADHD-I with SDRT, Tau and the RT fluctuations examined in the slow and fast conditions only marginally reduced in magnitude and remained significant; ADHD-HI remained significantly associated with SDRT, Slow-5, Slow-4, Slow-3 and Slow-2, with almost unchanged regression coefficients, but not with Tau, despite only a small reduction in the strength of the association (Table 2). When controlling for the effect of the ADHD traits, SCI was no longer significantly associated with SDRT in the slow condition and the associations of RRBI with RTV measures in the slow condition remained non-significant, with the regression coefficients for both associations being reduced (Table 2). As analyses indicated a specific association with ADHD traits only, we did not perform the planned post-hoc hierarchical regression testing for the additive effect of ADHD and ASD traits.

### Effect of ADHD and ASD Traits on RTV Changes across Conditions

**Fig. 1:**
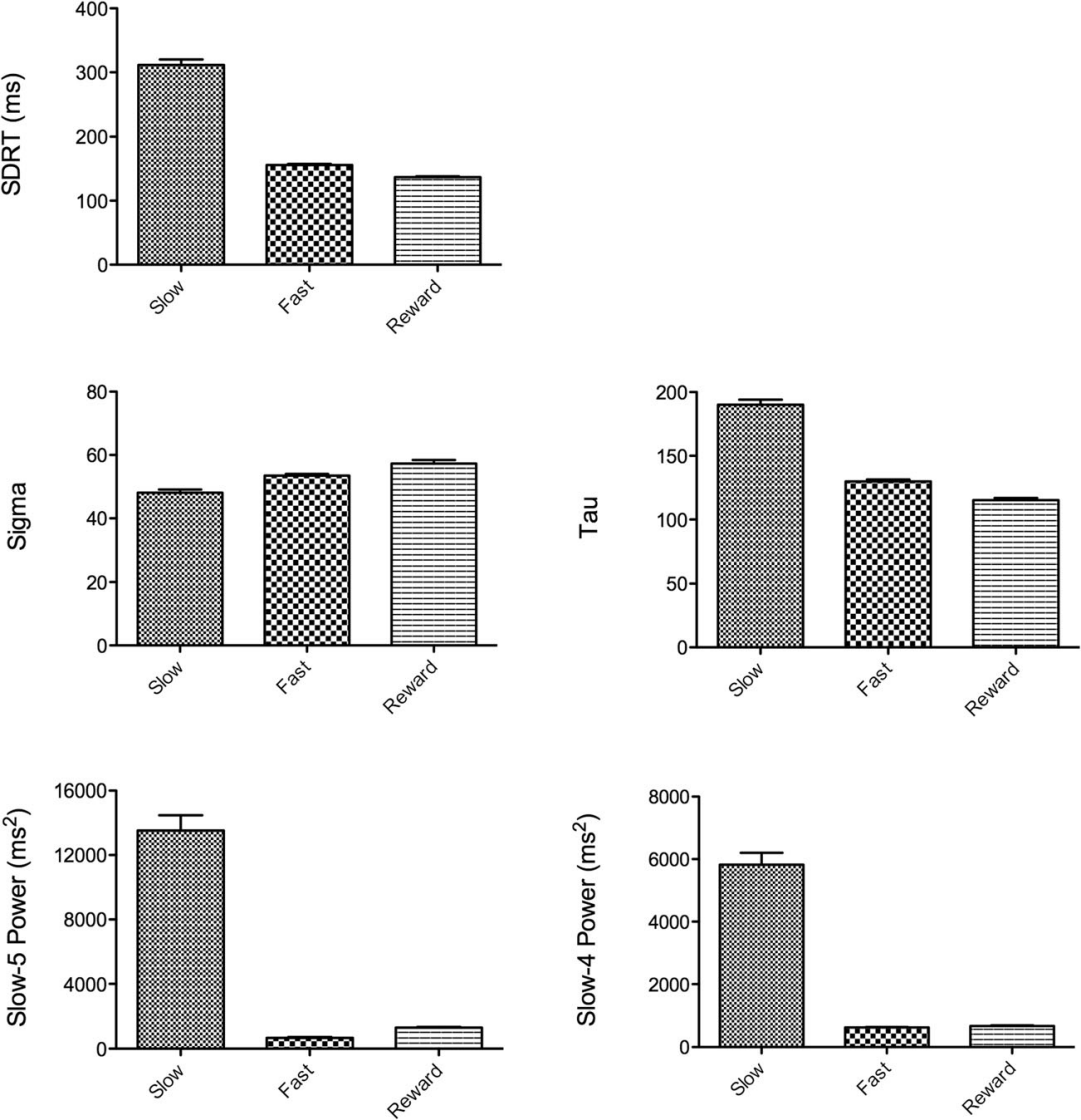
Mean (SE) of the standard deviation of reaction time (SDRT), Sigma, Tau, Slow-5 and Slow-4 RT fluctuations in slow, fast and incentive conditions

Untransformed values of SDRT and all detailed RTV measures in the three task conditions are represented in Fig. 1. The interaction effects of ADHD and ASD traits with the change across conditions are the focus here but, for completeness, we also report the statistics on the main effects of condition on the examined measures in the supplementary materials. The main effect of condition was significant for all examined RTV measures (Supplementary Table S1).

#### SDRT

Although with a small difference in the magnitude of the regression parameters, ADHD-I, but not ADHD-HI, showed a significant interaction with the effect of condition on SDRT (Table 3). Follow-up examination of the separate interaction effects revealed that higher levels of ADHD-I traits were associated with greater SDRT reduction from baseline to the incentive condition than lower ADHD-I traits, as indicated by a significant ADHD-I x incentive interaction (Table 3; previously reported as an association between slow-incentive SDRT difference score and total ADHD symptom scores (Kuntsi et al. 2009)). We then examined the model’s predictive effects under incentives to assess whether the improvement following the introduction of incentives resulted in a different association between ADHD-I and RTV: the association between ADHD-I and SDRT was weaker and not significant in the incentive condition (β = 0.05; 95% CI = −0.01,0.10; *p* = 0.09). We did not find a significant interaction between condition and SCI or RRBI on SDRT (Table 3).

**Table 3:** Interaction effects emerging from the mixed effects models for the measures captured by all task conditions

Interaction effect	SDRT	Sigma	Tau	Slow-5	Slow-4
ADHD-I x condition (overall), F_(2, 1109)_	4.28*	0.00	8.45***	4.42*	3.96*
ADHD-I x slow-to-fast, β [95% CI]	−0.06 [−0.13, 0.00]	0.00 [−0.08, 0.08]	−0.07 [−0.14, 0.00]	−0.01 [−0.10, 0.03]	−0.03 [−0.10, 0.04]
ADHD-I x slow-to-incentive, β [95% CI]	−0.10 [−0.16, −0.03]***	0.00 [−1.3, 1.2]	−0.14 [−0.21, −0.08]***	−0.09 [−0.16, −0.03]**	−0.10 [−0.16, −0.03]**
ADHD-HI x condition (overall), F_(2, 1109)_	3.56	0.69	0.55	4.61*	2.39
ADHD-HI x slow-to-fast, β [95% CI]	−0.05 [−0.12, 0.02]	0.03 [−0.04, 0.11]	−0.02 [−0.09, 0.05]	−0.04 [−0.11, 0.02]	−0.04 [−0.11, 0.03]
ADHD-HI x slow-to-incentive, β [95% CI]	−0.09 [−0.16, −0.02]	−0.01 [−0.09, 0.06]	−0.04 [−0.11, 0.03]	−0.10 [−0.16, −0.03]**	−0.08 [−0.14, −0.01]
SCI x condition (overall), F_(2, 1109)_	0.55	1.97	1.34	0.24	0.31
SCI x slow-to-fast, β [95% CI]	−0.03 [−0.10, 0.03]	0.07 [−0.01, 0.15]	−0.04 [−0.11, 0.03]	0.00 [−0.07, 0.06]	−0.01 [−0.08, 0.06]
SCI x slow-to-incentive, β [95% CI]	−0.03 [−0.10, 0.04]	0.00 [−0.07, 0.08]	0.02 [−0.05, 0.09]	−0.02 [−0.08, 0.04]	−0.03 [−0.09, 0.04]
RRBI x condition (overall), F_(2, 1109)_	0.08	0.71	4.56*	0.25	0.07
RRBI x slow-to-fast, β [95% CI]	−0.01 [−0.08, 0.06]	0.04 [−0.04, 0.11]	−0.02 [−0.09, 0.05]	−0.02 [−0.08, 0.05]	−0.01 [−0.08, 0.06]
RRBI x slow-to-incentive, β [95% CI]	−0.01 [−0.08, 0.06]	−0.01 [−0.08, 0.07]	0.08 [0.01, 0.15]*	−0.02 [−0.08, 0.04]	−0.01 [−0.08, 0.06]

*ADHD-I*, inattention; *ADHD-HI*, hyperactivity-impulsivity; *RRBI*, repetitive-restricted behaviours and interests; *SCI*, social-communication impairments; *SDRT*, standard deviation of RT

*p < 0.05; **p < 0.01; ***p < 0.001

#### Sigma

No significant trait x condition interactions emerged for either the ADHD or ASD traits on Sigma (Table 3).

#### Tau

**Fig. 2:**
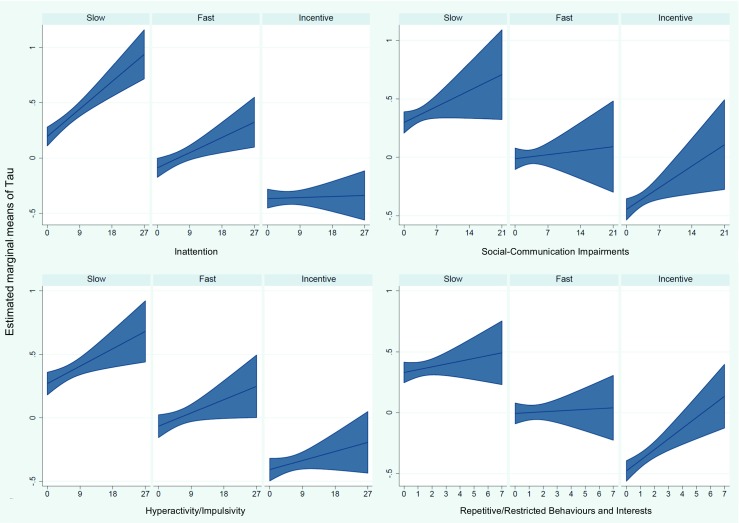
Relationship of the ex-Gaussian Tau with ASD and ADHD traits in slow, fast and incentive conditions. Means and 95% CIs are based on estimated marginal means of Tau as a function of mean centered ADHD and ASD traits, corrected for age and sex

For Tau, we found a significant main interaction of condition with ADHD-I and RRBI, but not with ADHD-HI or SCI (Table 3). Increasing levels of ADHD-I significantly predicted a greater Tau reduction from baseline to the incentive condition only, while a positive RRBI x incentive interaction indicated smaller reduction or potentially worsening of Tau with increasing RRBI under incentives (Table 3, Fig. 2). Additionally, Tau was not associated with ADHD-I (β = 0.01; 95% CI = −0.05,0.06; *p* = 0.84) but was positively, significantly associated with RRBI under incentives (β = 0.10; 95% CI = 0.05,0.17; *p* < 0.0001).

#### Slow-5

For the Slow-5 RT fluctuations, we found a main trait x condition interaction with both ADHD traits: increasing levels of both ADHD traits were associated with a greater reduction in Slow-5 from slow to incentive condition only (Table 3). Further, ADHD-I and ADHD-HI were not associated with Slow-5 in the incentive condition (ADHD-I: β = 0.03; 95% CI = −0.01,0.08; *p* = 0.18; ADHD-HI: β = 0.01; 95% CI = −0.04,0.07; *p* = 0.58). No significant interaction emerged between SCI or RRBI and condition.

#### Slow-4

For Slow-4 RT fluctuations, we found a significant main interaction between condition and ADHD-I, but not with ADHD-HI; no significant interaction between condition and SCI or RRBI emerged (Table 3). An increasing level of ADHD-I was associated with greater decrease of Slow-4 from slow to incentive condition only (Table 3). Additionally, ADHD-I was not associated with Slow-4 under incentives (β = 0.04; 95% CI = −0.02,0.09; p = 0.18).

In post-hoc analyses, testing the effects of each trait by controlling for the subdomains of the other trait, interactions between the ADHD and ASD traits remained substantially unchanged (Supplementary Table S2).

## Discussion

We show that, beyond what first appears a shared neurocognitive impairment of increased RTV between ADHD and ASD traits, specificity of this feature to ADHD traits emerges under closer inspection. Investigating frequency and ex-Gaussian RTV subcomponents in a large population sample of children, we found, first, that the refined RTV components were linked to ADHD traits and not to ASD traits. Second, although both ADHD and ASD social-communication traits were associated with the overall measure of SDRT, the association with ASD social-communications trait disappeared when controlling for ADHD traits, while association with ADHD traits remained when controlling for ASD traits. Third, a reward-induced improvement in RTV measures, indicating malleability, was only observed in relation to ADHD traits.

We found that the ex-Gaussian Tau was uniquely related to inattention and that the periodic RT fluctuations in slow and fast cycles showed specificity to inattention and hyperactivity-impulsivity. These findings are in line with the majority of prior studies on clinically diagnosed samples reporting increased amplitudes in these measures in participants with ADHD, but not those with ASD (Adamo et al. 2013; Johnson et al. 2007; Tye et al. 2016), although with modest effect sizes, as is common in general population samples where the use of the full range of scores detects modest effects. One previous study reported, however, elevated ex-Gaussian and frequency parameters in children diagnosed with ASD and those with comorbid ASD-ADHD but not in those with ADHD only (Geurts et al. 2008). As compared to the shorter duration (3-min) of the task used in Geurts et al. (2008), our tasks of ~8-min and ~9-min might have captured slower patterns of responses typically observed in relation to ADHD in longer tasks (Adamo et al. 2013; Johnson et al. 2007; Tye et al. 2016). The persistent association of Tau with inattention and the lack of an association of Tau with hyperactivity-impulsivity when controlling for ASD traits further suggest a closer association of this ex-Gaussian measure with inattention, and support previous hypotheses that the ultra-long, rare RTs captured by Tau may reflect lapses of attention (Leth-Steensen et al. 2000; West et al. 2002). Future work is warranted to further our understanding of how ASD traits affect the relationship of RTV measures with hyperactivity-impulsivity, and the pathophysiology underlying the detailed RTV measures. So far, only preliminary evidence has emerged for a direct association between the refined RTV measures and neural impairments, showing that relatively fast cycles in RTV parallel fluctuations in neural markers of attention allocation in healthy individuals (Adamo et al. 2015).

The lack of a significant association of Sigma with the behavioural traits in our study contrasts with three previous reports of elevated Sigma in ADHD and co-morbid ADHD-ASD in clinical samples (Geurts et al. 2008; Hervey et al. 2006; Tye et al. 2016), but parallels the results of another study that could not differentiate children with ADHD and control children using this index (Leth-Steensen et al. 2000). Such inconsistency across studies can be viewed in light of the results of a large meta-analysis of case-control studies on RTV in ADHD, which found that Sigma discriminates between ADHD and control groups with smaller effect sizes than Tau (Kofler et al. 2013). Increased RTV in individuals with ADHD is therefore more likely to reflect variability of the rare, abnormally long RTs, captured in Tau, rather than fluctuations in the normally distributed RTs, captured in Sigma (Kofler et al. 2013).

The only shared impairment between the ADHD and ASD traits, observed in the overall RTV measure of SDRT, was no longer associated with ASD social-communication difficulties when controlling for either ADHD trait, but remained significantly associated with both ADHD subdomains when controlling for ASD traits. The association of high SDRT in relation to ASD traits may therefore be explained by co-occurring ADHD symptoms, supporting previous evidence from clinical samples (Karalunas et al. 2014).

The investigation of the malleability of the high RTV provides another angle on potential specificity. Most previous research on the malleability of RT fluctuations has focused on the overall measure of SDRT in relation to ADHD (Cheung et al. 2017; Hervey et al. 2006; James et al. 2016). Conversely, Tye et al. (2016) extended this approach to ex-Gaussian RTV measures in an ADHD-ASD comparison, finding that the greater improvement of both overall and ex-Gaussian RTV measures under fast-incentive conditions were specific to ADHD, as such improvements were not observed in children with ASD only. Our findings confirm this observation in a population-based sample and further extend the findings to the frequency measures: when rewards were given, SDRT, Tau and the Slow-4 RT fluctuations decreased (i.e., improved) in relation to inattention, and Slow-5 RT fluctuations improved in relation to both ADHD subdomains, while no such effects emerged for the ASD traits. Together with prior evidence that cognitive performance is optimised in individuals with ADHD with the introduction of incentives (Andreou et al. 2007; Kofler et al. 2013; Kuntsi et al. 2009; Tye et al. 2016; Uebel et al. 2010), these findings support theories of atypical reward processes in ADHD, which in turn might help understand the neurobiology underlying the disorder (Luman et al. 2010). With our current and previous (Kuntsi et al. 2013b) results pointing to stronger associations of the RTV improvements with inattentive than hyperactive-impulsive traits, our findings further motivate future exploration of the neural correlates of RTV fluctuations in both ADHD subdomains. In our analyses, effects of faster event rate did not reach significance. We have previously reported how, in relation to ADHD diagnosis and trait, rewards tend to lead to a slightly greater SDRT improvement than fast event rates (Kuntsi et al. 2009; Uebel et al. 2010). Yet we have shown using quantitative genetic model-fitting analyses that both manipulations measure, to a large extent, the same underlying process (Kuntsi et al. 2013a). Further, jittered stimulus presentation has also previously shown to improve RTV measured as the ex-Gaussian Tau in children with ADHD (Lee et al. 2015), suggesting that response preparation may not be optimized with fast yet consistent event rates.

Our findings on the lack of positive effects from reward in relation to ASD traits are in agreement with reports of children with ASD benefiting less (Delmonte et al. 2012) or not at all (Scott-Van Zeeland et al. 2010) from the introduction of monetary rewards in other cognitive impairments compared to controls. For the clinician, these findings emphasise how, in the design of treatment protocols for attention impairments in children with neurodevelopmental disorders, different approaches may work with children with ASD than in children with ADHD, as the use of monetary or token incentives likely not have the expected reinforcing effects or might even worsen cognitive performance in these children. While we observed no association between ASD traits and improvement in the RTV measures following either rewards or a faster event rate, ASD restricted-repetitive behaviours and interests were significantly associated with a worsening in Tau following rewards. In reviewing this result, we consider emerging findings of aberrant temporal processing in children with ASD, who might integrate different stimuli into one single event over a longer window than normal controls (Foss-Feig et al. 2010). Accordingly, we tentatively speculate that an impaired integration of stimuli (i.e., incentives and target stimuli) might interfere with the processing of target stimuli, generating more variable responding. This motivates further investigations into ASD traits that use, for example, the high temporal resolution of electroencephalography to disentangle the neural basis of the effects of rewards on Tau.

The current study has some limitations. Behavioural ratings on ASD traits were collected approximately one year later than ADHD ratings and cognitive data, therefore potentially reducing the magnitude of observed associations of RTV measures with ASD traits. However, while in children with diagnosed ASD significant age-related increases in repetitive behaviours have been reported (Richler et al. 2010), autism traits have been reported to be stable over time in the general population (Gotham et al. 2012), limiting the potential effect of a lag between parent report and cognitive testing. The CAST scale provides a symptom count of the social-communication difficulties and the restricted-repetitive behaviours and interests, which may be a suboptimal measure of the full range of ASD traits in a population sample. Future research could benefit from examining the relationship of RTV indices with measures that capture the severity, rather than the presence or absence, of the ASD symptoms. An additional limitation is that the current study only focused on a population-sample of twins aged 7–10 years. Twins may not be representative of the general population in terms of mental health problems, as it has been suggested that twins might have an increased risk for ADHD compared to singletons (Levy et al. 1996). However, other studies have found little or no evidence for such differences (Gjone and Novik 1995; Robbers et al. 2010; Simonoff et al. 1997), thus this unlikely affects the interpretation of our results. Nevertheless, future research will need to establish the generalizability of our findings across a wider age range.

In sum, in an investigation of ADHD and ASD traits in a large population sample of children, detailed ex-Gaussian and frequency RTV indices, as well as reward-induced improvements in the RTV measures, show specificity to ADHD traits. As reports of increased RTV are not limited to ADHD and ASD (Kofler et al. 2013), our findings support the application of the ex-Gaussian and frequency approaches, and of reward manipulations, to further cross-disorder investigations. For the clinician planning effective behavioural interventions, our findings indicate that attentional fluctuation in children with high ASD traits may be due to co-occurring ADHD traits and emphasise how the effectiveness of rewards does not generalise from ADHD to ASD traits.

## Electronic supplementary material

ESM 1 (DOCX 15 kb)

ESM 2 (DOCX 15 kb)

ESM 3 (DOCX 19 kb)
