# Supplementary material for: Ex-Gaussian, Frequency and Reward Analyses Reveal Specificity of Reaction Time Fluctuations to ADHD and Not Autism Traits
Source: J Abnorm Child Psychol. 2018 Jul 19;47(3):557–67. doi: 10.1007/s10802-018-0457-z (PMC6397137; doi:10.1007/s10802-018-0457-z)
Supplement: Supplementary file 1 — (DOCX 15 kb) [file 10802_2018_457_MOESM1_ESM.docx]

**SUPPLEMENT 1**

**Sample and Procedure**

The participants were recruited from the Twins’ Early Development Study (TEDS) (Trouton et al. 2002) a birth cohort study which invited parents of all twins born in England and Wales during 1994-1996 to enrol. The TEDS families are representative of the UK population with respect to parental occupation, education and ethnicity (Oliver and Plomin 2007). TEDS families were invited to take part if they fulfilled the following SAIL project inclusion criteria: twins’ birthdates between September 1, 1995, and December 31, 1996; lived within a feasible travelling distance from the research centre; White European ethnic origin (to reduce population heterogeneity for molecular genetic studies); recent participation in TEDS, as indicated by return of questionnaires at either 4- or 7-year data collection point; no extreme pregnancy, perinatal difficulties, specific medical syndromes, chromosomal anomalies or epilepsy; not participating in other current TEDS substudies; and not on stimulant or other neuropsychiatric medications. Of the 1,230 suitable families contacted, 672 families (55%) agreed to participate. Thirty-two individual children were subsequently excluded due to: IQ < 70, epilepsy, obsessive compulsive disorder, autism or other neurodevelopmental disorder, illness during testing or placement on stimulant medication for ADHD.

Participants were invited to our research centre for a cognitive assessment, where ratings on the Conners’ scale were collected from parents. Participants completed the Wechsler Intelligence Scales for Children, Third Edition (WISC-III) (Wechsler 1991). The vocabulary, similarities, picture completion and block design subtests from the WISC-III were used to obtain a prorated estimate of the child’s IQ (Sattler 1992). Children’s IQs ranged from 70 to 158 (mean = 109.34, SD = 14.72). The families visited the research centre for the assessments. Two examiners assessed the twins simultaneously in separate testing rooms. The tasks were administered as part of a more extensive testing session, which in total lasted approximately 2.5-h.

**REFERENCES**

Oliver, B. R., & Plomin, R. (2007). Twins' Early Development Study (TEDS): a multivariate, longitudinal genetic investigation of language, cognition and behavior problems from childhood through adolescence. *Twin Res Hum Genet, 10*(1), 96-105.

Sattler, J. M. (1992). Assessment of children: WAIC-III and WPPSI-R Supplement. San Diego: Jerome M Sattler.: San Diego.

Trouton, A., Spinath, F. M., & Plomin, R. (2002). Twins early development study (TEDS): a multivariate, longitudinal genetic investigation of language, cognition and behavior problems in childhood. *Twin Res, 5*(5), 444-448, doi:10.1375/136905202320906255.

Wechsler, D. (1991). Wechsler Intelligence Scale for Children (3rd edn). *The Psychological Corporation: London*.
