# Supplementary material for: Ex-Gaussian, Frequency and Reward Analyses Reveal Specificity of Reaction Time Fluctuations to ADHD and Not Autism Traits
Source: J Abnorm Child Psychol. 2018 Jul 19;47(3):557–67. doi: 10.1007/s10802-018-0457-z (PMC6397137; doi:10.1007/s10802-018-0457-z)
Supplement: Supplementary file 2 — (DOCX 15 kb) [file 10802_2018_457_MOESM2_ESM.docx]

| Supplementary Table S1. Main effect of condition emerging from the mixed effects models for the measures captured by all task conditions. | | | | | | |
| --- | --- | --- | --- | --- | --- | --- |
|  | **SDRT** | **Sigma** | **Tau** | **Slow-5** | **Slow-4** | |
| **Condition effect (overall), F_(2, 1109)_** | 395.81*** | 42.29*** | 208.90*** | 704.22*** | | 402.07*** |
| *slow-to-fast, β [95% CI]* | -0.67 [-0.74, -0.60]*** | 0.34 [0.26, 0.42]*** | -0.36 [-0.43, -0.29]*** | -1.21 [-1.27, -1.14]*** | | -0.90 [-0.96, -0.83]*** |
| *slow-to-incentive, β [95% CI]* | -0.92 [-0.99, -0.86]*** | 0.26 [0.19, 0.35]*** | -0.72 [-0.80, -0.62]*** | -0.78 [-0.84, -0.71]*** | | -0.79 [-0.87, -0.73]*** |
| Note: results of the regression models including age and sex as covariates. ***p<0.001 | | | | | | |
