# Supplementary material for: Ex-Gaussian, Frequency and Reward Analyses Reveal Specificity of Reaction Time Fluctuations to ADHD and Not Autism Traits
Source: J Abnorm Child Psychol. 2018 Jul 19;47(3):557–67. doi: 10.1007/s10802-018-0457-z (PMC6397137; doi:10.1007/s10802-018-0457-z)
Supplement: Supplementary file 3 — (DOCX 19 kb) [file 10802_2018_457_MOESM3_ESM.docx]

| Supplementary Table S2. Effect of ADHD and ASD traits on RTV change across task conditions, controlling for the other trait. | | | | | | | | |  |
| --- | --- | --- | --- | --- | --- | --- | --- | --- | --- |
|  | **SDRT** | **Sigma** | **Tau** | **Slow-5** | **Slow-4** | | | | |
| **Interaction effect** |  |  |  |  |  | | | | |
| **ADHD-I x condition (overall), F_(2, 1109)_** | 4.28* | 0.00 | 8.44*** | 4.43* | | 3.96^ | |  |  |
| *ADHD-I x slow-to-fast, β [95% CI]* | -0.06 [-0.13, 0.00] | 0.00 [-0.08, 0.08] | -0.07 [-0.14, 0.00] | -0.03 [-0.10, 0.03] | | -0.03 [-0.10, 0.04] | |  |  |
| *ADHD-I x slow-to-incentive, β [95% CI]* | -0.10 [-0.16, -0.03]*** | 0.00 [-0.08, 0.08] | -0.14 [-0.21, -0.08]*** | 0.09 [-0.16, -0.03]** | | -0.10 [-0.16, -0.03] | |  |  |
| **ADHD-HI x condition *(overall), F_(2, 1109_****_)_* | 3.56 | 0.69 | 0.55 | 4.62* | | 2.40 | |  |  |
| *ADHD-HI x slow-to-fast, β [95% CI]* | -0.05 [-0.12, 0.02] | 0.03 [-0.04, 0.11] | -0.02 [-0.09, 0.05] | -0.04 [-0.11, 0.02] | | -0.04 [-0.11, 0.03] | |  |  |
| *ADHD-HI x slow-to-incentive, β [95% CI]* | -0.09 [-0.16, -0.02] | -0.01 [-0.09, 0.06] | -0.04 [-0.11, 0.03] | -0.10 [-0.16, -0.03]** | | -0.08 [-0.14, -0.01] | |  |  |
| **SCI x condition (overall)*, F_(2, 1109)_*** | 0.55 | 1.98 | 1.33 | 0.24 | | 0.32 | |  |  |
| *SCI x slow-to-fast, β [95% CI]* | -0.03 [-0.10, 0.03] | 0.07 [-0.01, 0.15] | -0.04 [-0.11, 0.03] | 0.00 [-0.07, 0.06] | | -0.01 [-0.08, 0.06] | |  |  |
| *SCI x slow-to-incentive, β [95% CI]* | -0.03 [-0.10, 0.04] | 0.00 [-0.07, 0.08] | 0.02 [-0.05, 0.09] | -0.02 [-0.08, 0.04] | | -0.03 [-0.10, 0.04] | |  |  |
| **RRBI x condition (overall), F_(2, 1109)_** | 0.08 | 0.70 | 4.56* | 0.25 | | 0.07 | |  |  |
| *RRBI x slow-to-fast, β [95% CI]* | -0.01 [-0.08, 0.06] | 0.04 [-0.04, 0.11] | -0.02 [-0.09, 0.05] | -0.02 [-0.08, 0.05] | | -0.01 [-0.08, 0.06] | |  |  |
| *RRBI x slow-to-incentive, β [95% CI]* | -0.01 [-0.08, 0.06] | -0.01 [-0.08, 0.07] | 0.08 [0.01, 0.15]* | -0.02 [-0.08, 0.04] | | -0.01 [-0.08, 0.06] | |  |  |
| ADHD-I: inattention; ADHD-HI: hyperactivity-impulsivity; RRBI: repetitive-restricted behaviours and interests; SCI: social-communication impairments; SDRT: standard deviation of RT; ^p=0.05; *p<0.05; **p<0.01; ***p<0.001. | | | | | | |  |  |  |
